# Effect of dexpanthenol on glycerol-induced acute kidney injury by targeting the PGC-1α/SIRT3 pathway

**DOI:** 10.1007/s00210-025-04071-5

**Published:** 2025-03-25

**Authors:** Fadimana Koyuncu, Filiz Alkaya Solmaz, Kanat Gulle, Ilter Ilhan, Muhammet Yusuf Tepebasi, Eyyup Sabri Ozden, Pakize Kirdemir

**Affiliations:** 1 Department of Anaesthesiology and Reanimation, Faculty of Medicine, Suleyman Demirel University, Cunur, 32260 Isparta, Turkey; 2 Department of Histology-Embryology, Faculty of Medicine, Suleyman Demirel University, Isparta, Turkey; 3 Department of Biochemistry, Faculty of Medicine, Suleyman Demirel University, Isparta, Türkiye; 4 Department of Genetic, Faculty of Medicine, Suleyman Demirel University, Isparta, Türkiye

**Keywords:** Acute kidney injury, Dexpanthenol, Oxidative stress, PGC-1α, Rhabdomyolysis, SIRT-3

## Abstract

Rhabdomyolysis (RM) can lead to life-threatening myoglobinuric acute kidney injury (AKI). Despite various treatment modalities for AKI, their effectiveness remains limited. Dexpanthenol (DEX) is an antioxidant, anti-inflammatory, and anti-apoptotic agent with demonstrated protective effects on various tissues. The current study aimed to investigate the protective effects and genetic mechanisms of DEX in AKI due to glycerol-induced RM. Thirty-two female Wistar Albino rats weighing between 250–300 g were allocated into four groups of eight rats each. The control group was given five days of intraperitoneal saline. The RM group was treated with an intramuscular injection of 8 ml/kg of 50% glycerol solution. The RM + DEX group was administered an intramuscular injection of 8 ml/kg of 50% glycerol solution and an intraperitoneal injection of 500 mg/kg DEX for five days, starting one hour after glycerol administration. The DEX group was treated with an intraperitoneal injection of 500 mg/kg DEX for five days. On the sixth day, rats were sacrificed and kidney tissues were taken. Histopathological analyses were performed on kidney tissue. Biochemical analyses were performed on kidney tissue and blood to evaluate kidney function and oxidative stress (BUN, creatinine, urea, CK, LDH, cystatin C, TAS, TOS, MDA, and CAT). Additionally, PGC-1α and SIRT-3 gene expression levels in kidney tissue were determined by qRT-PCR. All biomarkers significantly increased in the RM group. DEX treatment significantly reduced urea and creatinine levels. The increase in TOS levels and OSI in the RM group was significant compared to the control group, DEX treatment significantly reversed these effects. The RM and RM + DEX groups exhibited RM and nephropathy. Histopathological analysis revealed improvements in the RM + DEX group compared to the RM group. DEX treatment increased the expression of *PGC-1α* and *SIRT-3* in the RM + DEX group. Histopathological and biochemical improvements, including reduced kidney damage and oxidative stress, were observed with DEX treatment and was associated with increased expression of the *PGC-1α* and *SIRT-3* genes.

## Introduction

Acute kidney ınjury (AKI) is a sudden decline in kidney function occurring over a few hours or days, which can happen with or without pre-existing chronic kidney disease. This condition is marked by elevated levels of urea and creatinine in the blood and a reduced glomerular filtration rate (GFR) (Kondabolu et al. 2023). Identifying kidney damage before a decrease in GFR is crucial for diagnosis. Although kidney damage begins very early, serum creatinine levels do not increase in ARF unless GFR falls to a certain level. Therefore, serum creatinine and urea increases are insufficient biomarkers for diagnosing ARF. Cystatin C is less affected by chronic diseases, nutritional changes, and changes in muscle mass compared to serum creatinine. For this reason, it has been shown to be more sensitive than serum creatinine in the early diagnosis of ARF. Markers such as cystatin C and neutrophil gelatinase-associated lipocalin are used for early detection of AKI (Wen and Parikh 2021). Despite various antioxidant therapies for AKI due to rhabdomyolysis (RM), no routine clinical treatment is currently effective.

Rhabdomyolysis results from muscle tissue breakdown and necrosis, leading to the release of components like electrolytes, creatine kinase, and myoglobin into the bloodstream (Cabral et al. 2020). Etiological factors include trauma, intense exercise, infections, drugs, and toxins (Młynarska et al. 2022). Myoglobin released from damaged muscle cells causes hemoglobinuria and myoglobinuria, triggering renal vasoconstriction, tubular obstruction, tubular injury, and lipid peroxidation, contributing to AKI (Gupta et al. 2021).

Understanding the pathophysiology of AKI has involved studying various animal models. Glycerol-induced AKI models feature myoglobinuria, acute tubular necrosis, and renal vasoconstriction (Semenovich et al. 2022; Izuwa et al. 2009).

Free oxygen radicals are short-lived, unstable chemical products with unpaired electrons (Kondabolu et al. 2023; Di Meo and Venditti 2020). Excessive production of these radicals and disruption of antioxidant systems cause damage to cellular lipids, DNA, and proteins. Lipid peroxidation involves the oxidation of membrane phospholipids, resulting in the formation of peroxide derivatives (Su et al. 2019). Malondialdehyde (MDA), a byproduct of lipid oxidation caused by superoxide radicals (SORs), correlates well with the degree of lipid peroxidation (Zorova et al. 2016). Glutathione peroxidase (GPx) and catalase (CAT) are enzymatic antioxidants that protect cells from the harmful effects of SORs (Özcan et al. 2015).

Mitochondrial dysfunction plays a major role in AKI pathogenesis. PGC-1α is a transcriptional coactivator that regulates mitochondrial biogenesis and function in various tissues, including the kidneys. Increased expression of *PGC-1α* enhances the number of mitochondria, aerobic respiration capacity, and ATP concentration in proximal tubular cells, supporting repair and recovery from oxidative damage (Rasbach and Schnellmann 2007).

Sirtuins, including *SIRT3*, are NAD^+^-dependent deacetylases with diverse cellular functions. *SIRT-3*, located in the mitochondrial matrix, contributes to ATP production by modulating electron transport chain complexes (Rahman et al. 2014). *SIRT-3* is thought to play a critical role in AKI pathogenesis by regulating mitochondrial dynamics, oxidative stress, and apoptosis (Yang et al. 2016). Activated by *PGC-1α*, *SIRT-3* enhances oxidative stress defense and mitochondrial recovery (Clark and Parikh 2021).

Dexpanthenol (DEX), an alcohol analog of pantothenic acid (vitamin B5), exhibits antioxidant activity by blocking lipid peroxidation (Ozcan et al. 2024; Cagin et al. 2016; Karadag et al. 2015). In studies involving patients with RM injury, DEX treatment has been associated with significant reductions in hospital stay and dialysis requirements compared to controls (Martin-Lorenzo et al. 2017). Although the exact mechanism of DEX’s protective effect in RM remains unclear, it is hypothesized to possess antioxidant and anti-inflammatory properties that may mitigate muscle and kidney damage (Üremiş et al. 2024).

To date, there is insufficient research on DEX’s protective effects and genetic mechanisms in AKI induced by RM. This study aimed to fill this gap by exploring the protective effects of DEX and underlying mechanism in a rat model of AKI induced by RM, employing biochemical, histopathological, and with a specific focus on mitochondrial dynamics regulated by PGC-1α and SIRT-3.

## Materials and methods

### Animals and study design

Thirty-two adult female Wistar albino rats (250–300 g) were kept under standard conditions (21–22 °C, 60 ± 5% humidity) with a 12:12 light/dark cycle and were fed a standard commercial diet (Korkuteli Yem, Antalya, Türkiye).

Rats in the control group (*n* = 8) received 1 ml of saline intraperitoneally for five days (Wen and Parikh 2021). The RM group (*n* = 6) had their fluid intake restricted 12 h prior to the experiment and was administered 8 ml/kg of 50% glycerol solution intramuscularly (1 ml per leg) (Pinar et al. 2019; Singh et al. 2012; Song et al. 2020). Two rats died during the procedure. Rats in the RM + DEX group (*n* = 6) also had their fluid intake restricted 12 h before the experiment. They received 8 ml/kg of 50% glycerol solution intramuscularly and 500 mg/kg DEX intraperitoneally for five days, starting 1 h after glycerol administration. Two rats in this group also died. Rats in the DEX group (*n* = 8) received 500 mg/kg DEX intraperitoneally for five days.

**Fig. 1:**
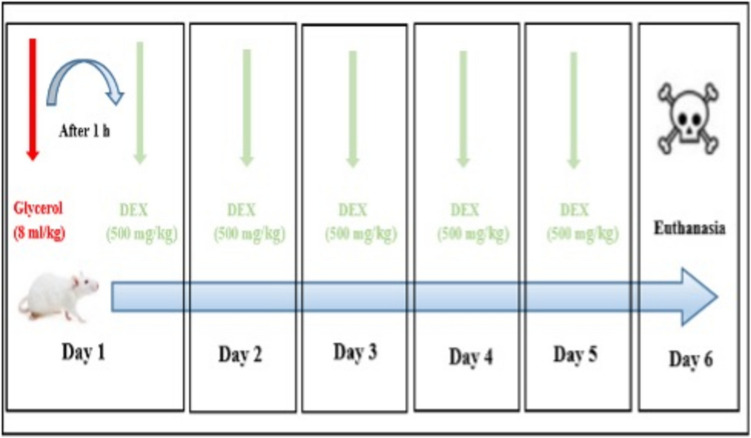
Experimental design of the study. The experimental design, including the application times of Glycerol and DEX as well as the sacrifice time points. The study consisted of the control, DEX, RM + DEX, and DEX groups, each containing eight animals. DEX: Dexpanthenol

On the sixth day, rats were anesthetized with 80–100 mg/kg ketamine (Ketalar; Pfizer, Istanbul, Türkiye) and 8–10 mg/kg xylazine (XylazinBio 2%; Bioveta, Plc, Ivanovice na Hané, Czech Republic). After anesthesia, rats were sacrificed, and blood samples were collected from the inferior vena cava via abdominal incision. Kidney samples were stored at −20 °C for biochemical analysis and −80 °C for quantitative reverse transcription-polymerase chain reaction (qRT-PCR) analysis, while the remaining tissue was fixed in 10% buffered formalin for histopathological examination (Fig. 1).

### Histopathological examination

Kidney samples were fixed in 10% buffered formalin. Following routine histological processing, tissues were embedded in paraffin. Sections of 5 µm thickness were cut using a rotary microtome (Leica RM2155; Leica Microsystems, Wetzlar, Germany), stained with hematoxylin and eosin (H&E), mounted on slides, and examined under a light microscope (Olympus CX21, 40X, Tokyo, Japan).

### Measurement of blood parameters

Blood samples were collected in gel tubes and centrifuged at 3,000 rpm for 10 min. The serum was stored at −80 °C until analysis. Urea, CK, LDH, and creatinine levels were measured using a Beckman Coulter AU5800 analyzer (Beckman Coulter, Inc., Brea, CA, USA) with compatible kits (Beckman Coulter Urea/Urea Nitrogen Analysis Kit, Beckman Coulter Creatinine Analysis Kit). Cystatin C was measured using an ELISA kit (BT LAB, Zhejiang, China) specific for rats.

### Kidney CAT activity

Catalase activity was measured using the Aebi method on a UV-1601 Shimadzu Spectrophotometer (Shimadzu Corp., Kyoto, Japan). Results are expressed as kU/g protein (Aebi 1984).

### Kidney MDA activity

Malondialdehyde levels were determined using the Draper and Hadley method for TBARS with double heating. An 8360 μmol/L stock solution was prepared with 40% ethanol and 50 μl TEP. From this, 0.1 ml was diluted with 40% ethanol to a final volume of 20 ml to create Standard 1. Additional standards were prepared through serial dilutions.

From the supernatant of tissue homogenates, 0.5 ml was mixed with 2.5 ml of 10% TCA. The same process was applied to the standards and blanks. The samples were vortexed, heated in boiling water for 15 min, then quickly cooled. The samples were centrifuged at 5000 rpm for 10 min. Two milliliters of the upper supernatant were mixed with 1 ml of 0.67% TBA. The mixture was vortexed and heated in boiling water for 15 min. After cooling, the absorbance was measured against the blank at 532 nm using the UV-1601 Shimadzu spectrophotometer (Erel 2005). MDA levels in kidney tissue are reported as µmol/g protein.

### Measurement of oxidative stress parameters

The levels of total oxidant status (TOS) and total antioxidant status (TAS) in the kidney tissue supernatants were measured using commercial kits (Rel Assay Diagnostics, Gaziantep, Türkiye) with a spectrophotometric method on a Beckman Coulter AU5800 auto-analyzer (Erel 2005, 2004). The measured TAS and TOS values were used to calculate the oxidative stress index (OSI) using the following formula: OSI = TOS / TAS × 100.

### Quantitative reverse transcription-polymerase chain reaction (qRT-PCR)

According to the manufacturer’s instructions, total RNA was extracted from tissues using the GeneAll Ribospin RNA isolation kit (cat. no: 301–001) (GeneAll Biotechnology Co. Ltd., Seoul, Korea). The amount and purity of the RNA samples were assessed using a NanoDrop device (Thermo Scientific NanoDrop™; Thermo Fisher Scientific Inc., Waltham, MA, USA) before cDNA synthesis. For cDNA synthesis, 20 µl of reverse transcriptase reaction mixture was prepared in PCR tubes for each sample. cDNA synthesis was carried out in a thermal cycler following the A.B.T.™ cDNA Synthesis Kit (Atlas Biotechnologies, Ankara, Türkiye) protocol.

Real-time PCR amplification was performed using SYBR Green Master Mix (Atlas Biotechnologies, Ankara, Türkiye) (cat. no: Q04-01–05) on a CFX96 device (Bio-Rad, Hercules, CA, USA) according to the manufacturer’s instructions. Specific primers for the amplification of *SIRT-3*, *PGC-1α* (primer sequences available upon request), and glyceraldehyde-3-phosphate dehydrogenase (GAPDH) were designed. cDNA samples were analyzed in triplicate for each PCR run. GAPDH expression was used for normalization, and gene expression was assessed relative to controls using the comparative ΔΔCt method.

### Statistical analysis

Statistical analyses between groups were performed using the IBM SPSS Statistics v.23 software (IBM, Armonk, NY, USA). One-way analysis of variance (ANOVA) with post hoc LSD (least significant difference) test was used for biochemical, qRT-PCR, histopathological, and immunohistochemical analyses [3]. The Kruskal-Wallis test was employed to determine significant differences between groups. Statistical significance was set at *p* < 0.05.

## Results

### Histopathological results

**Fig. 2:**
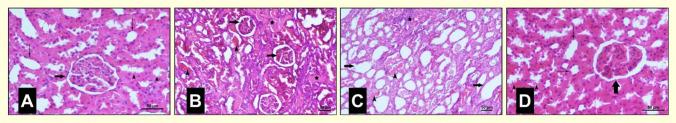
Microscopic appearance of the kidneys. **A** Normal kidney tissue in the control group Proximal tubule (arrowhead), distal tubule (thin arrow), and glomeruli (thick arrow). **B** In the RM group, there is fibrosis and atrophy in the glomeruli (thick arrow), extensive tubular damage with eosinophilic material in some tubules and damaged tubular epithelium (arrowhead), and intense protein accumulation in other tubules (star). **C** In the RM + DEX group, the number of dilated collecting tubules (arrow), eosinophilic material accumulation and intense protein accumulation (cast) observed in tubules (arrowhead), and fibrosis in the interstitial area (star) appear reduced compared to the RM group. Compared to the RM group, tubular epithelia were found to be preserved (arrow). **D** In the DEX group, kidney tissue shows normal distal tubules (thin arrow) and glomeruli (thick arrow), with occasional detachment of proximal tubular epithelium (arrowhead). H&E; scale bar = 50 µm; DEX: dexpanthenol, RM: rhabdomyolysis

Histopathological examination revealed extensive tubular damage, significant eosinophilic material accumulation and pronounced interstitial fibrosis in kidney sections from the RM group, with dense fibrosis observed in the interstitial space. Fibrosis and atrophy were noted in glomeruli, along with widespread tubular damage. DEX treatment reduced inflammatory cell infiltration and other pathological findings in the RM + DEX group. Normal tissue histology was observed in the control and DEX groups (Fig. 2).

The histopathological evaluation was performed using a semi-quantitative scoring method. The severity of inflammatory cell infiltration, vascular congestion, tubular dilatation, and degeneration of tubular epithelial cells was graded as follows:: (-) score: no structural changes; ( +) score: mild structural changes; (+ +) score: moderate structural changes; (+ + +) score: severe structural changes (Table 1).

**Table 1:** Histopathological comparison of all groups

Group	Inflammatory cell infiltration	Vascular congestion	Tubular dilatation	Degeneration of tubular epithelial cells
Group Control	**-**	**-**	**-**	**-**
Group RM	** + + + **	** + + + **	** + + + **	** + + + **
Group RM + DEX	** + **	** + **	** + **	** + **
Group DEX	**-**	**-**	**-**	**-**
Control vs RM	***p* < 0.05**	***p* < 0.05**	***p* < 0.05**	***p* < 0.05**
Control vs (RM + DEX)	*p* < 0.05	*p* < 0.05	*p* < 0.05	*p* < 0.05
Control vs DEX	***p* > 0.05**	***p* > 0.05**	***p* > 0.05**	***p* > 0.05**
RM vs (RM + DEX)	*p* < 0.05	*p* < 0.05	*p* < 0.05	*p* < 0.05
RM vs DEX	***p* < 0.05**	***p* < 0.05**	***p* < 0.05**	***p* < 0.05**
DEX vs (RM + DEX)	*p* < 0.05	*p* < 0.05	*p* < 0.05	*p* < 0.05

### Biochemical results

**Fig. 3:**
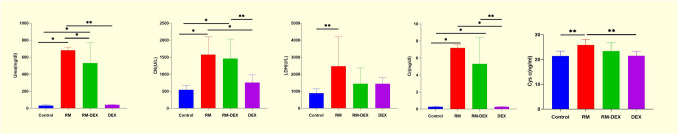
Urea, CK, LDH, Cr and Cys c levels. Values are presented as mean ± SD. RM: Rhabdomyolysis. DEX: Dexpanthenol, CK: Creatinine Kinase, Cr: Creatinine, LDH: Lactate Dehydrogenase, Cys c: Cystatine c *p < 0.05, **p < 0.01

To assess kidney function, urea, CK, LDH, and creatinine levels were measured biochemically. Compared to the control group, all biomarkers significantly increased in the RM group (*p* < 0.001, *p* < 0.001, *p* = 0.004, and *p* < 0.001, respectively). DEX treatment significantly reduced urea and creatinine levels (p < 0.028 and *p* < 0.034). Urea, CK, and creatinine levels were significantly lower in the DEX group compared to the RM group (*p* < 0.001, *p* = 0.001, and *p* < 0.001, respectively) (Fig. 3). Compared to the control group, cystatin C levels were significantly increased in RM group (*p* < 0.002).

**Fig. 4:**
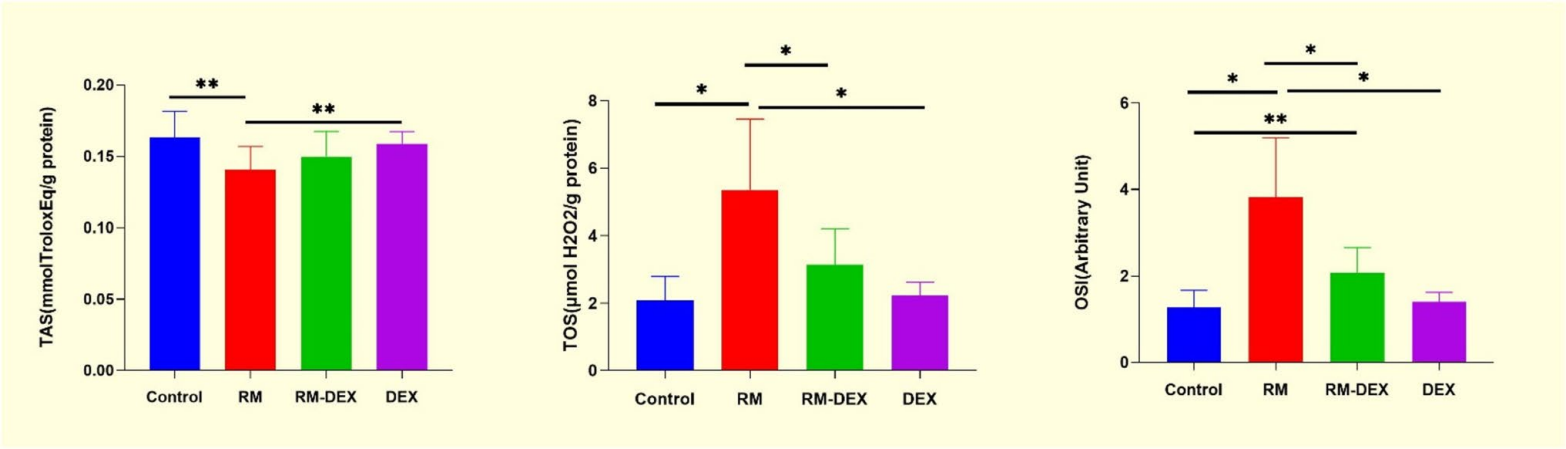
TAS, TOS, OSI levels. Values are presented as mean ± SD. RM: Rhabdomyolysis. DEX: Dexpanthenol, TAS: Total Antioxidant Status, TOS Total Oxidant Status:, OSI: Oxidative Stress İndex *p < 0.001, **p < 0.01

To indicate oxidative stress in kidney tissues, TOS and TAS levels were measured, and OSI was calculated. The increase in TOS levels and OSI in the RM group was significant compared to the control group (*p* < 0.001 for both). DEX treatment significantly reversed these effects (*p* < 0.001 for both) (Fig. 4).

**Fig. 5:**
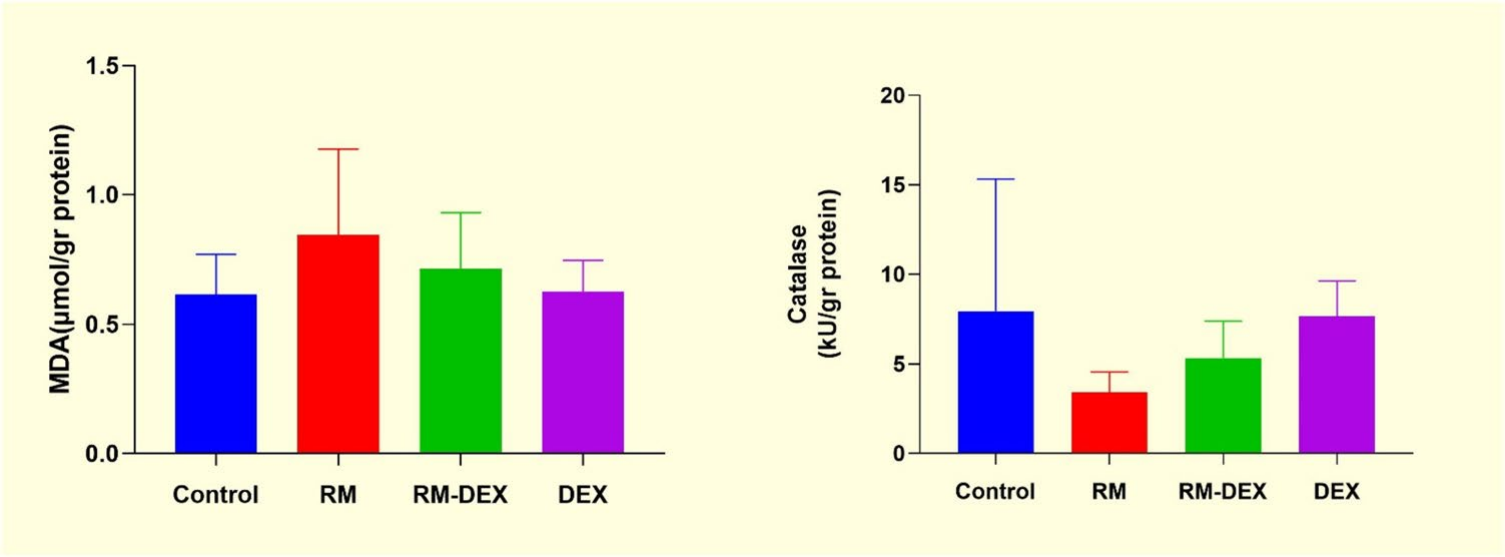
MDA, Cataşase levels. Values are presented as mean ± SD. RM: Rhabdomyolysis. DEX: Dexpanthenol, MDA: Malondialdehyde

Malondialdehyde and CAT levels were measured in kidney tissue. No statistically significant differences were observed between the groups (Fig. 5).

**Fig. 6:**
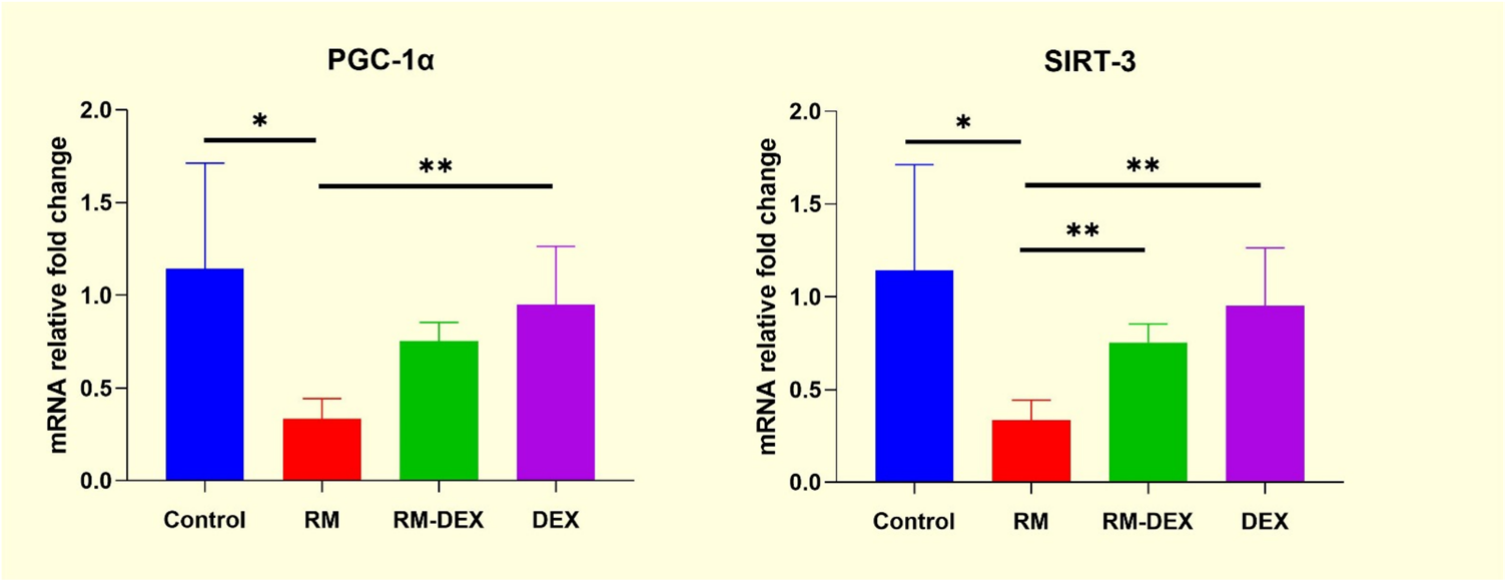
PGC-1α and SIRT-3 mRNA relative fold change. Values are presented as mean ± SD. RM: Rhabdomyolysis. DEX: Dexpanthenol, PGC-1α: Peroxisome proliferator-activated receptor-gamma coactivator—1alpha. SIRT-3: Sirtuin-3 *p < 0.001, **p < 0.01

*PGC-1α* and *SIRT-3* levels were assessed using qRT-PCR. Compared to the control group, *PGC-1α* and *SIRT-3* levels were significantly reduced in the RM group (*p* < 0.001 and *p* < 0.01, respectively). While DEX treatment reversed the decrease in *SIRT-3* levels, it did not result in a significant increase in *PGC-1α* levels (*p* > 0.05). In the DEX group, *PGC-1α* and *SIRT-3* levels were significantly higher compared to the RM group (*p* = 0.004 and *p* < 0.01, respectively) (Fig. 6).

## Discussion

The analyses conducted in this study demonstrated that renal damage caused by RM induced by an 8 ml/kg dose of intramuscular glycerol in rats was improved histopathologically and by decreasing *PGC-1α* and *SIRT-3* gene expression through five days of intraperitoneal 500 mg/kg DEX application.

Histopathological examinations have revealed that necrotic cell death and inflammation in AKI can lead to parenchymal cell loss, tubular epithelial degeneration, and tubulointerstitial fibrosis with a reduced number of tubules (Martin-Sanchez et al. 2017; Karabacak et al. 2016). Previous studies have also shown that DEX has histopathological protective effects by reducing leukocyte infiltration in rat kidney tissue affected by AKI due to RM (Semenovich et al. 2022; Ozden et al. 2023). In our study, significant differences in histological changes, such as inflammatory cell infiltration and tubular epithelial degeneration, were observed in the DEX group compared to the RM group (*p* < 0.05), aligning with similar studies in the literature. We hypothesize that DEX’s histopathological protective effect may be attributed to its suppressive effect on inflammatory cells.

The pathogenesis of AKI due to crush syndrome involves fluid retention by damaged myocytes and ischemic acute tubular necrosis ATN development due to renal hypoperfusion. Myoglobinuria and the formation of myoglobin plugs lead to tubular toxicity (Oto and Sever 2023). Elevated blood urea nitrogen and serum creatinine levels indicate renal vasoconstriction in AKI (Młynarska et al. 2022). Our study observed increased levels of urea, CK, creatinine, and LDH in the AKI caused by glycerol-induced RM, with improvements noted following DEX treatment. Cystatin C, an endogenous marker frequently used to determine GFR, is less affected by changes in muscle mass compared to serum creatinine (Izuwa et al. 2009). In our study, the difference in creatinine levels was possibly due to changes in muscle mass. Increased cystatin C levels in the RM group indicated AKI development in kidney tissue. DEX exhibited renal protective effects; however, its influence on cystatin C appears limited or may necessitate prolonged administration.

The increase in SOR production plays a crucial role in tubule cell damage in AKI (Tomsa et al. 2019). Elevated levels of TOS and OSI in the RM group indicated increased oxidative stress in the kidney tissue. DEX’s protective effect appears to be primarily mediated through modulation of systemic oxidative balance. The absence of significant changes in MDA, catalase, or TAS levels between the RM and RM + DEX groups suggests limited direct influence on specific antioxidant pathways, despite a notable reduction in TOS and OSI.

PGC-1α is a transcriptional coactivator that plays a crucial role in the regulation of mitochondrial biogenesis and function, rather than serving as a direct biomarker of mitochondrial damage (Abu Shelbayeh et al. 2023). A study investigating mitochondrial functions in kidney tissue with AKI found increased *SIRT-3* gene expression during renal damage (Zhou et al. 2022). The same study also showed that treatment improved the AKI condition by activating the *PGC-1α/Nrf/SIRT-3* pathway involved in mitochondrial biogenesis. Our study found that decreased expression of *PGC-1α* and *SIRT-3* genes in the RM group was associated with mitochondrial damage in the affected kidney tissue, which aligns with existing literature. Increased expression of *PGC-1α* and *SIRT-3* genes in the RM + DEX group was linked to DEX’s protective effect in the renal damage recovery mechanism. The results suggest that DEX treatment can prevent damage mechanisms triggered by glycerol-induced AKI through *PGC-1α* and *SIRT-3* signaling, thus protecting kidney structure in rats. Further studies could explore the application of glycerol and DEX on other tissues and their effects. Understanding the expression mechanisms of *PGC-1α* and *SIRT-3*, key regulatory genes of mitochondrial biogenesis, may be crucial for developing effective treatment modalities in AKI and clarifying the action mechanism of DEX.

The study’s limitations include the lack of detailed antioxidant analyses (such as superoxide dismutase, nitric oxide, GPx, and glutathione reductase) and the failure to evaluate DEX’s protective effect against renal damage using parameters like TNF-α and caspase 3. Another limitation is that the protective effect of dexpanthenol on renal failure caused by rhabdomyolysis has not been studied in male rats. Male rats can be used in future studies.

## Conclusions

DEX provides significant protection against AKI due to glycerol-induced RM in rats. Histopathological and biochemical improvements, including reduced kidney damage and oxidative stress, were observed with DEX treatment. Enhanced expression of *PGC-1α* and *SIRT-3* further supports DEX’s protective role in mitochondrial function and oxidative stress defense. These findings suggest that DEX is a promising therapeutic agent for managing AKI associated with RM.

## Data Availability

All source data for this work (or generated in this study) are available upon reasonable request.
